# Durability of the Bond between CFRP and Concrete Exposed to Thermal Cycles

**DOI:** 10.3390/ma12030515

**Published:** 2019-02-08

**Authors:** Shuai Liu, Yunfeng Pan, Hedong Li, Guijun Xian

**Affiliations:** 1 School of Civil Engineering and Architecture, Zhejiang Sci-Tech University, Hangzhou 310018, China; sliu_2008@163.com (S.L.); lihedong@zstu.edu.cn (H.L.); 2 School of Civil Engineering, Harbin Institute of Technology, Harbin 150001, China; gjxian@hit.edu.cn

**Keywords:** CFRP-to-concrete, bond, fracture energy, thermal cycles

## Abstract

The bond between carbon fiber reinforced polymer (CFRP) and concrete is significantly and adversely affected by thermal cycles in air and water. In the present study, the effects of thermal cycles in air or water on the bond performance between CFRP and concrete were examined. A single-lap shear test was adopted to evaluate the performance of the CFRP–concrete bond. A number of 270 thermal cycles in air increased the interfacial fracture energy of the CFRP plate– and CFRP sheet–concrete by 35% and 20%, respectively while 270 thermal cycles in water reduced the interfacial fracture energy of the CFRP plate– and CFRP sheet–concrete by 9% and 46%, respectively. Thermal cycles in water caused the failure mode to change from concrete cohesive failure to primer–concrete interfacial debonding. The failure modes of CFRP–concrete exposed to thermal cycles in air still occurred in concrete. A reduction factor for the CFRP–concrete structure for thermal cycles in water was proposed.

## 1. Introduction

Externally bonded carbon fiber reinforced polymer (CFRP) has become a popular technology for the rehabilitation of concrete structures. The technology depends on the integrity of the CFRP–concrete interfacial bond. A numerical and experimental study was adopted to investigate the short-term performance of FRP−concrete [1,2,3]. Although FRP composites are considered highly durable, the harsh environment may deteriorate the interfacial bond between CFRP and concrete [4]. A long-term cyclic temperature in the field results in the variation of temperature. It has been reported that the interfacial bond between CFRP and concrete is susceptible to low temperature, high temperature, thermal cycles, relative humidity, and freeze–thaw cycles [5,6]. Few studies have been conducted to investigate the effects of thermal cycles, relative humidity, and their combination on bond performance. Because a long service life is required for civil structures, the lack of data on the long-term durability data of CFRP–concrete bonds is concerning and needs to be addressed urgently.

The high and low temperatures induced by thermal cycles affect the interfacial failure of CFRP–concrete bonds [7]. The glass transition temperatures (*T*_g_) of construction adhesives are relatively low, generally ranging from 45 to 82 °C [8]. The elastic modulus, tensile strength, and ductility of the adhesives increase with increasing temperature (below *T*_g_) and curing time. Compared to CFRP sheet–concrete at 20 °C, the interfacial fracture energy at −10 °C decreased by 15%, whereas a 12% increase of interfacial fracture energy was reported at 40 °C (below *T*_g_) [7]. The coefficients of thermal expansion of concrete, resin, and CFRP are 10 × 10^−6^, 35 × 10^−6^, and 1 × 10^−6^ (1/°C), respectively [7,9]. The mismatch in the coefficient of thermal expansion results in the fatigue of interfacial stress between CFRP and concrete. The interfacial fracture energy increased by 26% with 35 thermal cycles from 30 to 40 °C [10]. This means that the small number of thermal cycles deteriorates the CFRP–concrete bond insignificantly. The increase in fracture energy is attributed to the effects of postcuring on the adhesive and FRP. With a further increase to 365 thermal cycles, the interfacial fracture energy decreased by 12% [10]. The fatigue of the interfacial stress plays a dominant role in degradation of interfacial bonds and causes microcracks between the adhesive layer–concrete interface. 

The moisture level plays a very important role in the effect of thermal cycles on the deterioration of CFRP–concrete performance [5,11,12]. On the one hand, the combination of water moisture and thermal cycles deteriorates the constituent materials. The water moisture in the concrete deteriorates the surface of the concrete owing to the frost action at low temperature (below zero). The damage degree is related to the water ingress into the concrete. The durability properties of concrete and other cementitious materials are directly related to their microstructure [13,14,15,16]. Therefore, the water uptake of the concrete depends on the concrete internal structures—e.g., void content and pore structure [17]. Some studies have shown that the concrete compressive strength varied insignificantly with 200 freeze–thaw cycles [18]. Other research has shown that the concrete compressive strength decreased by 86% with 50 freeze–thaw cycles in water [12]. On the other hand, the water moisture deteriorates the chemical bond between primer and concrete [19]. Water molecules between silica (concrete) and epoxy enlarge the distance between the molecules and disrupt the hydrogen bond [20]. As reported, the fracture energy of CFRP–concrete decreased by 62.8% after water exposure at 50 °C for 8 weeks [21]. Eighteen months of water immersion reduced the fracture energy by 68% for CFRP–high strength concrete with $f_{c}^{\prime }$ = 88.6 MPa [4].

Thermal cycles in air or water may cause variation in the CFRP–concrete failure modes under shear. Water immersion was reported to shift the failure modes of debonding from concrete cohesive fracture to interfacial debonding [4]. The deterioration of the primer–concrete bond is more significant than the tensile strength of the concrete. The failure modes of CFRP–concrete under thermal cycles in water still occur in concrete owing to severe degradation of the concrete compressive strength [12]. The thermal cycles in air enhance the interfacial bond and the postcuring of the adhesive. The failure modes of the thermal cycles in air were associated with a 2–5 mm thick concrete layer attached to debonding FRP [10].

As discussed, the presence of water moisture causes the differences in the performance of CFRP–concrete between thermal cycles and freeze–thaw cycles. The present study investigates the effects of thermal cycles in air or water on the bond behavior of a CFRP–adhesive–concrete system. The CFRP type and thermal cycles in air and water were considered in the present study. The evolution of the constituent materials and the interfacial bond between CFRP and concrete was studied under the conditions of thermal cycles in air and water. This paper also sheds light on the effect of water moisture on the bond failure mechanism at the interface region by degradation at the interface.

## 2. Experimental

### 2.1. Raw Materials

A pultruded CFRP plate and sheet were adopted in the present study. The fiber volume fraction of the pultruded CFRP plates was 67%, and its dimensions were 1.4 mm in thickness and 25 mm in width. The nominal thickness of the CFRP sheet was 0.167 mm. The fiber volume fraction of the sheet with a wet layup was 30%. The tensile test of the CFRP plate/sheet was carried out according to the ASTM D3039 method [22]. 

The concrete consisted of water, cement, sand, and gravel with a mass ratio of 0.43: 1.0: 1.29: 2.75. Concrete blocks (100 mm × 100 mm × 400 mm and 100 mm × 100 mm × 100 mm) were prepared and cured at 95% relative humidity (RH) for 3 months. The concrete compressive strength of 100 mm × 100 mm × 100 mm cubes was 55.4 MPa [23]. A modification coefficient (0.76) was adopted for the cylindrical compressive strength of concrete [23]. The mean cylindrical compressive strength of concrete ($f_{c}^{\prime }$) was 42.1 MPa. In the present study, the elastic modulus was determined using the ACI 318 procedure, according to the equation $E_{c}=4730\sqrt{f_{c}^{\prime }}$ in MPa [24]. The elastic modulus of the concrete was found to be 30.7 GPa.

### 2.2. Single-Lap Shear Test

**Figure 1:**
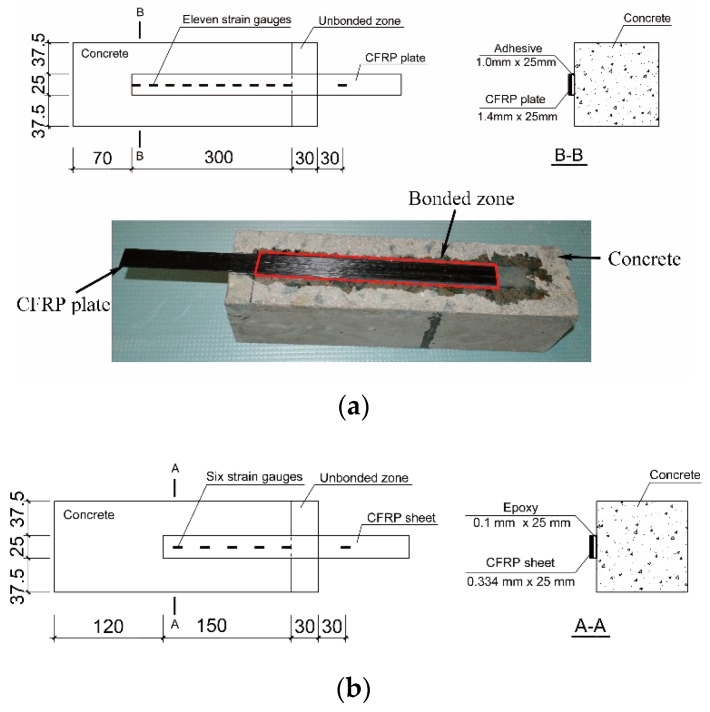
Details of carbon fiber reinforced polymer (CFRP) plate–concrete (CPC) (**a**) CFRP sheet–concrete (CSC) (**b**) (all units in mm).

Table 1 lists the constituent materials of CFRP–concrete. Epoxy and adhesive were selected in the present study. Figure 1 shows the dimensions of the CFRP–concrete specimens. The top layer of the cement paste on the bonding surface of the concrete was ground using a disk grinder. The ground surface was then cleaned with acetone. The primer was brushed onto the cleaned surface. To obtain an accurate measurement of the thickness of the adhesive layer for CFRP plate–concrete, 1 mm thick steel strips were placed between the CFRP plate and the concrete block, and the adhesive was brushed onto the surface of the primer. Subsequently, the pultruded CFRP laminate was attached to the adhesive layer in the longitudinal direction of the concrete block. To prepare the CFRP sheet–concrete, a CFRP sheet with the necessary saturant of epoxy was attached to the surface. The excess epoxy and the small air bubbles were removed using a squeegee. Teflon sheets were used to prevent adhesion between epoxy and concrete in the unbonded zone. All specimens were kept at room temperature for 1 month to ensure that the adhesive was well cured. Equation (1) was adopted to determine the effective bond length [25].

(1) $$L_{e}=\sqrt{\frac{E_{f}t_{f}}{\sqrt{f_{c}^{\prime }}}},$$

where $L_{e}$ is the effective bond length (mm); $E_{f}$ is the elastic modulus of FRP (GPa); $f_{c}^{\prime }$ is the concrete compressive strength (MPa); and $t_{f}$ is the thickness of FRP (mm). 

Using Equation (1), the effective bond lengths were determined to be 195 mm and 112 mm for CFRP plate–concrete and CFRP sheet–concrete, respectively. The sufficient bond lengths of 300 mm and 150 mm were applied for CFRP plate–concrete and CFRP sheet–concrete, respectively.

The CFRP strain along the centerline of the CFRP was measured with electrical resistance foil strain gauges spaced at 30 mm. The stain gauges of type BE 120-3AA (Zhonghang Electronic Measuring Instrument Co. Ltd., Hanzhong, China) were used. Six and eleven strain gauges were placed along the centerline of the CFRP sheet and CFRP plate, respectively, as shown in Figure 1.

**Figure 2:**
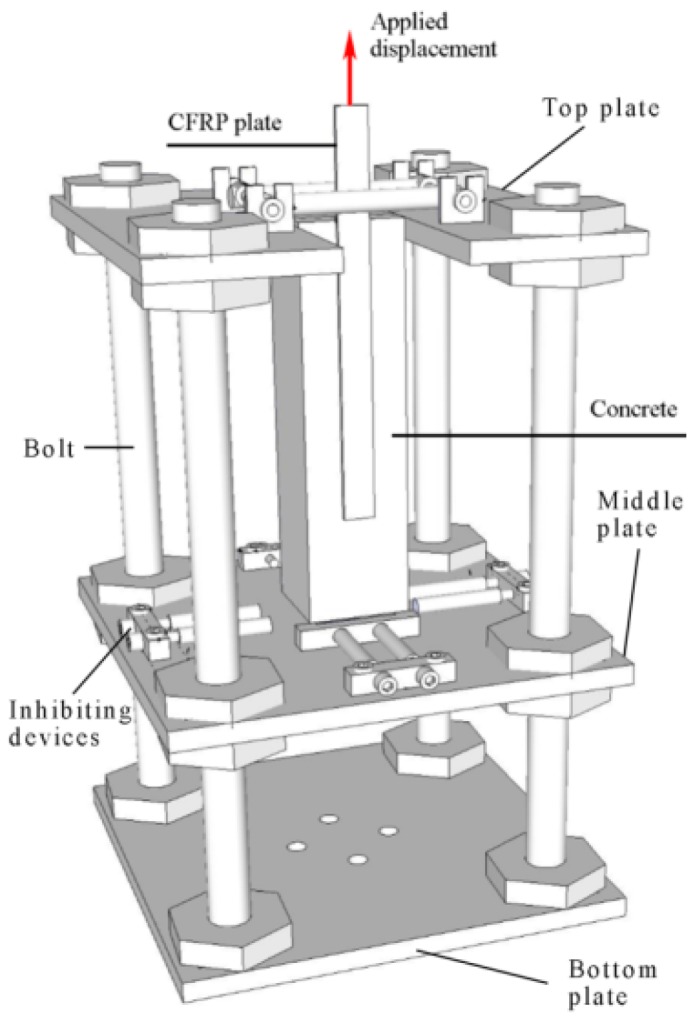
Single-lap shear test setup.

Figure 2 shows the single-lap shear test setup. The CFRP–concrete specimens were restricted by the top plate and the middle plate. Inhibiting devices were clamped to the sides of the specimen to restrict their translational and torsional movement. A displacement rate of 0.1 mm/min was controlled by an electronic universal testing machine equipped with a 100 kN loading cell.

### 2.3. Exposure Conditions

**Figure 3:**
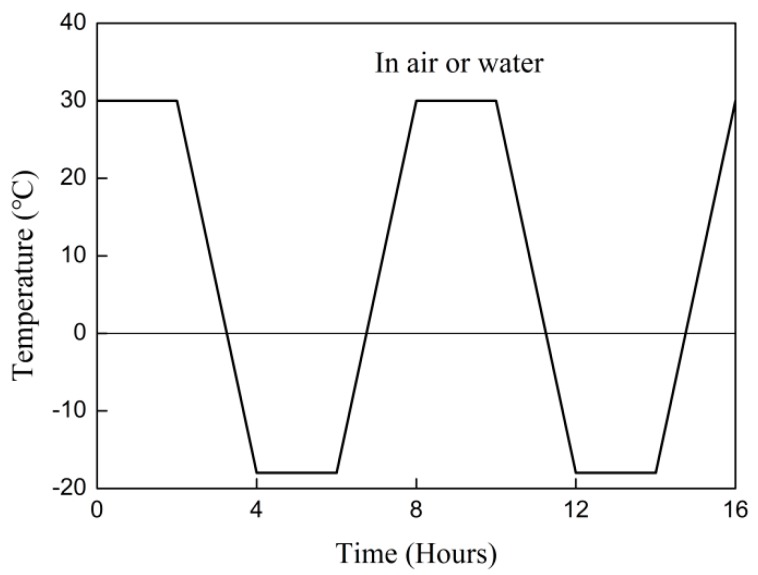
Freeze–thaw cycle parameters.

Figure 3 shows the temperature variation in the chamber during the high–low thermal cycles. The temperature was held constant at −18 and 30 °C for 2 h. The freezing from 30 to −18 °C and thawing from −18 to 30 °C was achieved within 2 h. Two conditionings were adopted. First, the specimens were immersed in water. Second, the specimens were exposed to thermal cycles in air.

## 3. Results and Discussion

### 3.1. Durability of Constituent Materials

**Figure 4:**
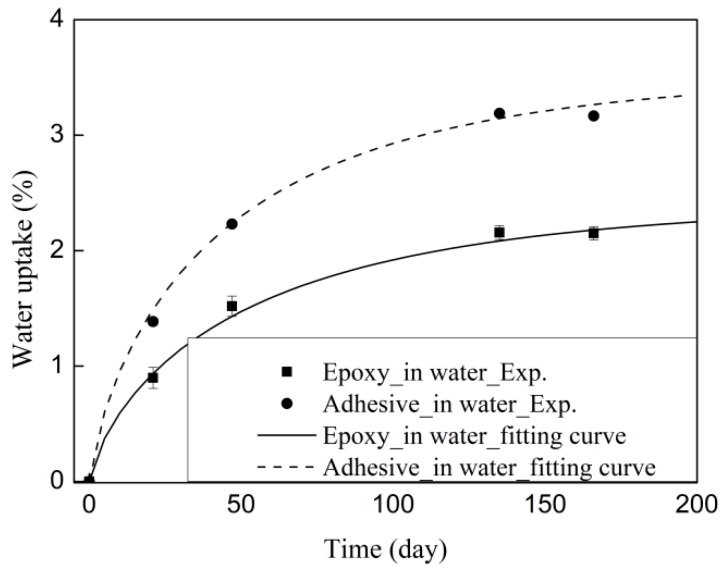
Water uptake curves of the epoxy.

The epoxy and adhesive with thermal cycles in air was assumed to be independent of moisture. The water uptake of the epoxy and adhesive was considered in water conditions. The moisture uptake of the epoxy and adhesive as a function of the square root of the exposure duration is shown in Figure 4. The weight gains of the epoxy and adhesive increase proportionally with the square root of the exposure duration before reaching saturation level, following Fickian’s law [26]. The equation of the Fickian law can be expressed as:

(2) $$M_{t}=M_{\infty }\left\{1-\exp\left[-7.3{\left(\frac{Dt}{t_{r}^{2}}\right)}^{0.75}\right]\right\},$$

where $M_{t}$ is the moisture uptake at time *t*, $M_{\infty }$ is the quasi-equilibrium moisture uptake, *t*_r_ is the thickness of the epoxy and adhesive (2 mm for the studied samples), and *D* and $M_{\infty }$ can be determined by Equation (2) and are listed in Table 2. 

The equilibrium moisture content of the adhesive is 1.5 times greater than that of the epoxy. The moisture uptake depends on the chemical structures of the epoxy system [27]. 

To characterize the moisture diffusion in CFRP, the diffusivity coefficient and the equilibrium moisture content of the CFRP sheet can be determined by [28]:

(3) DFRP=Dr(1−2vfπ),

(4) $$M_{\mathrm{FRP}}=M_{r}(1-v_{f}),$$

where $D_{\mathrm{FRP}}$ and $v_{f}$ are the diffusivity coefficient and fiber volume fraction of the CFRP, respectively, and $M_{\mathrm{FRP}}$ and $M_{r}$ are the equilibrium moisture contents of the CFRP and the corresponding matrix, respectively. The diffusivity properties of the CFRP determined by Equations (3) and (4) are listed in Table 3. The diffusivity coefficient of the CFRP sheet is 4.6 times greater than that of the CFRP plate. This means that the saturation of the CFRP plate requires more exposure time with the same thickness of the CFRP sheet and CFRP plate. The equilibrium moisture of the CFRP sheet is 2.1 times greater than that of the CFRP plate.

The variation of the mechanical properties of the epoxy and adhesive sample exposed to thermal cycles in air and water is listed in Table 4. The thermal cycles in water reduce the elastic modulus of the CFRP sheet and CFRP plate by 9% and 2%, respectively. The effects of the thermal cycles in air on the elastic modulus of the CFRP sheet and CFRP plate are insignificant. The degradation of CFRP is related to its hygrothermal stress and thermal stress. In the case of the thermal cycles in air, the effects of thermal stress on CFRP are insignificant. In the case of the thermal cycles in water, the coupling of water moisture and thermal cycles results in plasticization of the epoxy matrix and deterioration of the fiber–matrix bond [29]. 

The thermal cycles in air increase the elastic modulus of the epoxy and adhesive by 13% and 6%, respectively, whereas the thermal cycles in water reduce the elastic modulus of the epoxy and adhesive by 9% and 14%, respectively. The increase in elastic modulus is caused by the increases in the cross-link density of the epoxy and adhesive owing to postcuring. It has been reported that the enhancement of the elastic modulus has positive effects on the CFRP–concrete interfacial fracture energy [30].

### 3.2. Test Phenomena and Failure Modes

**Figure 5:**
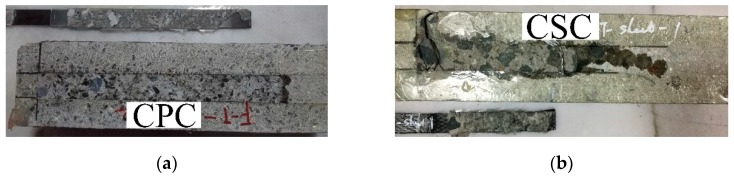

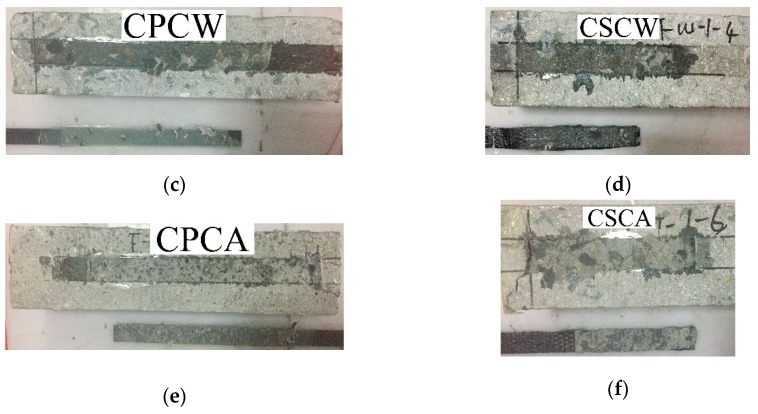
Failure mode of (**a**) control specimens of CFRP plate-concrete, (**b**) control specimens of CFRP sheet–concrete, (**c**) CFRP plate–concrete exposed to the thermal cycles in water, (**d**) CFRP sheet–concrete exposed to the thermal cycles in water, (**e**) CFRP plate–concrete exposed to the thermal cycles in air, (**f**) CFRP sheet–concrete exposed to the thermal cycles in air.

The failure modes of all specimens are listed in Table 5. Figure 5 shows the failure modes of the FRP–concrete bond. The failure modes of the control specimens occur in the concrete beneath the adhesive layer. A concrete block and many particles of fine aggregates were seen to be bonded to CFRP plates/sheets in the literature [5,31]. 

Thermal cycles in air result in peeled-off concrete. The failure modes are similar to those of the control specimens. Thermal cycles in air increase the bond between the primer and the concrete. This means that the internal stress induced by 270 thermal cycles in air insignificantly influences the interfacial bond.

In the case of thermal cycles in water, the failure modes shift from concrete cohesive failure to interfacial debonding between the primer and the concrete. This phenomenon has been reported in the case of CFRP–concrete in water [20,32]. The present failure mode of the interfacial debonding is different from the concrete cohesive failure of CFRP–concrete exposed to thermal cycles (−20 to 30 °C) in water [5]. Ref. [5] shows a 22% reduction of concrete compressive strength. In the present case, 270 thermal cycles in water reduced the compressive strength by 3.6%.

### 3.3. The Influence of the Exposure Conditions on the Interfacial Fracture Energy and Bond Stress–Slip Curve

The load capacity fluctuates with increased slip owing to the local failure of the concrete. To avoid fluctuation, the strain (ε) recorded by strain gauges can be expressed as a function of slip (s) as follows [30]:

(5) $$\varepsilon =f\left(s\right)=A\left(1-e^{-Bs}\right),$$

where *A* and *B* can be fitted by the ε−s curve of the experimental results. *A* is the maximum strain in the FRP with a sufficiently long bond length. The average maximum strain ($\varepsilon_{\max}$) at the peak load was determined by Equation (5). The slips were computed by integrating the strains measured on the surfaces of the CFRP. The detailed determination of slip was referred from Ref. [20].

The ultimate load capacity ($P_{u}$) can be obtained by Equation (6).

(6) $$P_{u}=E_{f}\cdot b_{f}\cdot t_{f}\cdot \varepsilon_{\max},$$

where $b_{f}$ and $t_{f}$ are the width and thickness of CFRP, respectively.

The interfacial fracture energy ($G_{f}$) can also be expressed as [30]:

(7) $$G_{f}=\frac{P_{u}^{2}}{2E_{f}t_{f}b_{f}^{2}},$$

**Figure 6:**
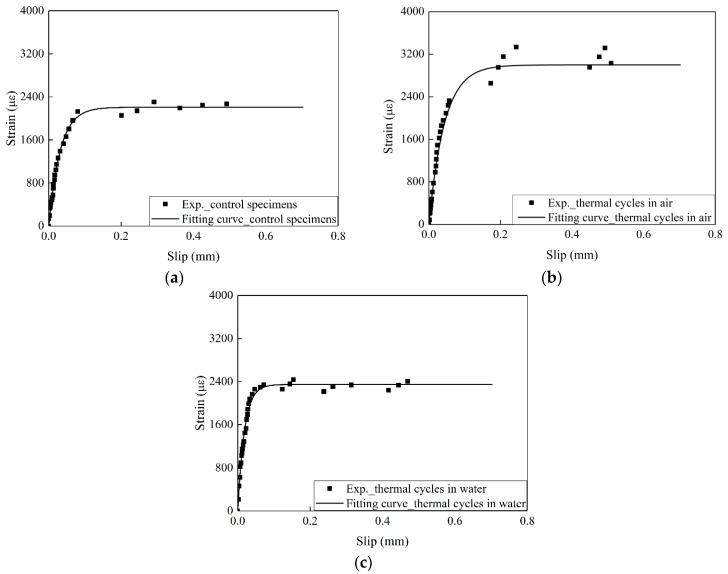
Regressed *ε*-*s* curve from the experimental results for CFRP plate–concrete with (**a**) control specimens, (**b**) thermal cycles in air, (**c**) thermal cycles in water.

Figure 6 shows the tested strain–slip results and the corresponding regressed fitting curves for the CFRP–concrete samples. *A* and *B* are determined using Equation (5), and *P*_u_ is determined using Equation (6). The values of *A* and *B*, the ultimate load capacity, and the interfacial fracture energy are listed in Table 5. 

In the case of the control specimens, the interfacial fracture energy of the CFRP plate–concrete is 55% larger than that of CFRP sheet–concrete. It has been reported that the interfacial fracture increases with FRP stiffness ($E_{f}t_{f}$) [30]. In the present study, the stiffness of the CFRP plate is three times that of the CFRP sheet. 

**Figure 7:**
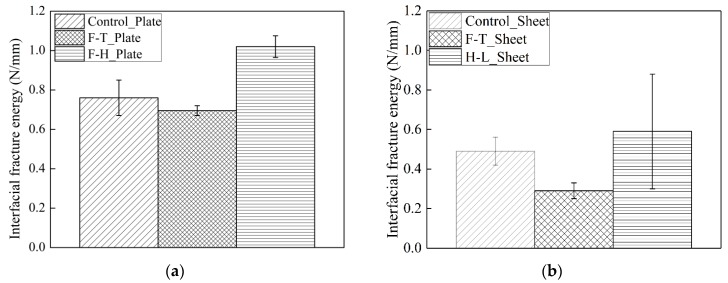
Interfacial fracture energy of CFRP plate–concrete (**a**) and CFRP sheet-concrete (**b**).

Figure 7 shows the variability of the interfacial fracture energy exposed to thermal cycles in air and water. Figure 7a indicates that the interfacial fracture energy of the CFRP plate–concrete increased by 35% owing to the thermal cycles in air, whereas the thermal cycles in water reduced the interfacial fracture energy by 9%. Figure 7b shows that the thermal cycles in air increased the interfacial fracture energy of the CFRP sheet–concrete by 20%, whereas a 41% reduction of the interfacial of the CFRP sheet–concrete was found under the thermal cycles in water conditions. The increase of fracture energy for both CFRP plate–concrete and CFRP sheet–concrete exposed to thermal cycles in air can be explained as follows. On the one hand, it is attributed to the enhancement of the interfacial bond between primer and concrete owing to the elevated temperature. On the other hand, the evaluated temperature increases the crosslink of the primer and the adhesive. This results in an increase of the elastic modulus in the primer and the adhesive. In the case of the thermal cycles in water, the decrease of the interfacial fracture energy results from the penetration of the water moisture at the primer–concrete interface. The chemical bond between primer and SiO_2_ (concrete) deteriorates significantly owing to the disruption of hydrogen bonding and the reduction of van der Waals forces [20,33].

Compared to a 9% reduction in the interfacial fracture energy of CFRP plate–concrete exposed to thermal cycles in water, the thermal cycles in water reduce the interfacial fracture energy of the CFRP sheet–concrete by 41%. The thickness and fiber volume fraction of the CFRP plate are 4.2 and 2 times those of the CFRP sheet, respectively. This means that the saturation time of the CFRP sheet is less than that of the CFRP plate, which indicates that the deterioration of the interfacial bond in CFRP sheet–concrete is more severe than that of CFRP plate–concrete. 

The increase in the interfacial fracture energy of CFRP plate–concrete is more than that of CFRP sheet–concrete under thermal cycles in air conditions. Thermal cycles in air induce the internal stress fatigue owing to the deformation of the adhesive and the primer. The deformation of the CFRP does not vary under the thermal cycles in water owing to very low thermal expansion coefficient of CFRP. The thickness of the adhesive layer (including primer and adhesive) provides the gradient of the deformation between CFRP and concrete. The 0.2 mm thickness of the adhesive layer in CFRP sheet– concrete results in greater internal stress. 

The bond stress–slip relationship can be expressed as a function of *A* and *B* [30]:

(8) $$\tau =A^{2}BE_{f}t_{f}\left(e^{-Bs}-e^{-2Bs}\right),$$

where τ and *s* are the bond stress (MPa) and the corresponding slip, respectively. 

**Figure 8:**
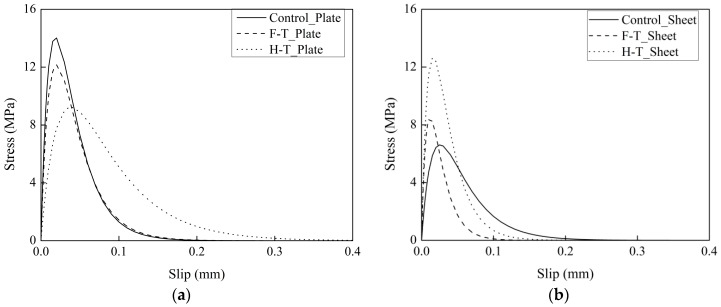
Bond stress–slip relationship of CFRP plate-concrete (**a**) and CFRP sheet-concrete (**b**).

The bond stress–slip curves determined by Equation (8) are shown in Figure 8. In the case of CFRP plate–concrete, the thermal cycles in air reduce the maximum bond stress owing to enhancement of the toughness of the adhesive layer at elevated temperatures. It has been reported that interfacial fracture energy is improved and maximum stress is reduced with increasing toughness of the adhesive layer [30]. Compared to the control specimens, the maximum stress of the specimens exposed to thermal cycles in water decreased owing to the deterioration of the interfacial bond between primer and concrete. In the case of CFRP sheet–concrete exposed to thermal cycles in air, a 92% increase in the maximum stress is attributed to the postcuring of the primer. The thermal cycles in water decrease the ductility of the interfacial bond.

### 3.4. The Influence of Exposure Conditions on the Interfacial Effective Bond Length

**Figure 9:**
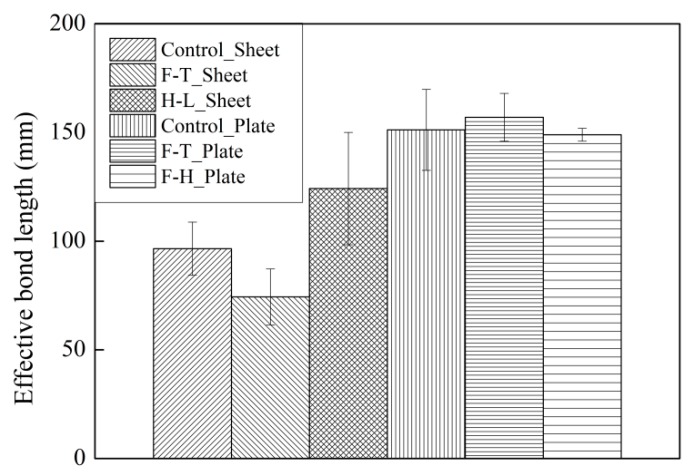
Variation of the effective bond length.

The effective bond length (*L*_e_) was determined by strain distribution along the CFRP. The detailed determination was described in Ref. [5,34]. The effects of the thermal cycles in air and water on the tested effective bond length are shown in Figure 9.

Figure 9 shows the insignificant effects of thermal cycles on the CFRP plate–concrete. In the case of the CFRP plate–concrete, the thermal cycles in water increased the effective bond length by 4%, whereas the effective bond length decreased by 1.5% owing to the thermal cycles in air. This phenomenon was reported in the CFRP plate–concrete exposed to the coupling of 90% relative humidity (RH) and thermal cycles [5]. 

The effects of thermal cycles in air and water on the effective bond length of CFRP sheet–concrete are significant. A 56% increase in the effective bond length was induced by the thermal cycles in air. Thermal cycles in air result in the postcuring of CFRP sheets. The effective bond length increases with increasing FRP stiffness according to Equation (1). In the case of CFRP sheet–concrete exposed to thermal cycles in water, a 23% reduction in the effective bond length of CFRP sheet–concrete results from the deterioration of the interfacial bond between primer and concrete. 

### 3.5. The Evolution of Fracture Energy Exposed to Thermal Cycles

As discussed above, the mechanism of the influence on the CFRP–concrete bond varies with the exposure environment. Table 6 lists the single-lap shear test data with thermal cycles in air and water collected from the literature [5,7,10,11,18,34]. 

**Figure 10:**
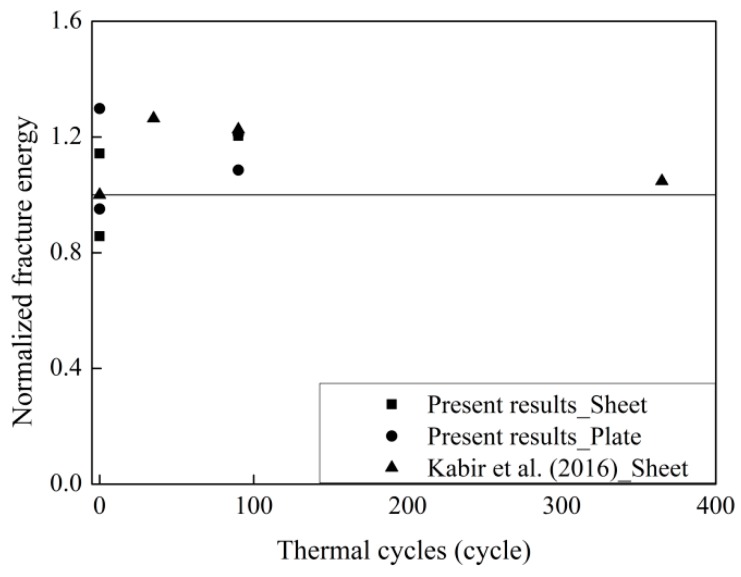
Effects of thermal cycles on the normalized fracture energy.

Only one dataset on the effects of thermal cycles in air on CFRP–concrete was found to correlate with the present study. Figure 10 shows the normalized fracture energy with the thermal cycles in air. The fracture energy of the aged specimens is distributed above the line of *y* = 1. This means that the thermal cycles in air increase the interfacial fracture energy. The fracture energy decreases by 15% with increasing thermal cycles in air from 90 to 365. This means that the short duration of thermal cycles has a positive influence on the fracture energy. The fatigue of the internal stress induced by the thermal cycles in air plays a role in the fracture energy with increasing thermal cycles.

**Figure 11:**
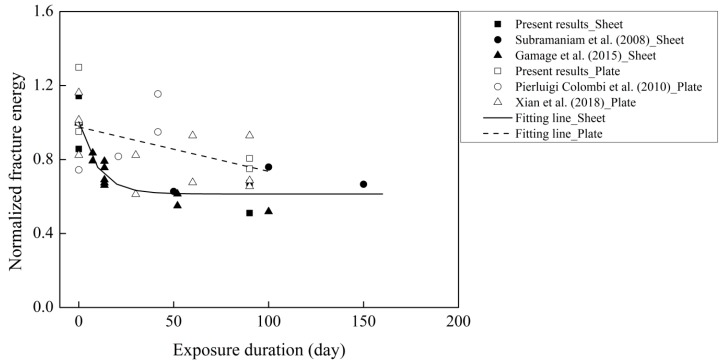
Effects of the exposure duration on the normalized fracture energy.

The collected data from Subramaniam et al. (2008), Xian et al. (2018), and Gamage et al. (2015) were adopted for correlation analysis with the present study of thermal cycles in water [5,11,34]. Canonical correlation analysis was performed using the commercial software SPSS to correlate the deterioration of fracture energy with the variation—e.g., concrete compressive strength and exposure duration [35]. The weak correlation with a correlation coefficient value of 0.011 indicates that the interfacial fracture energy is independent of the evolution of the compressive strength. The correlation coefficient value of 0.611 shows a strong correlation between the fracture energy and the exposure duration. Figure 11 shows the effects of the exposure duration on the normalized fracture energy. The normalized fracture energy decreases with increasing exposure duration. Compared to the CFRP plate, the reduction in the fracture energy of the CFRP sheet–concrete is more severe. The thickness and diffusivity coefficient of the CFRP sheet are smaller than those of the CFRP plate. The penetration of water moisture through the decreased thickness requires less time, resulting in greater deterioration of the CFRP sheet–concrete. 

The normalized fracture energy of both CFRP sheet–concrete and CFRP plate–concrete seems to reduce to a constant value. It has been reported that the bond binding energy due to Van der Waals interaction is reduced by 2/3, whereas the interlocking interaction does not vary with unlimited immersion time [20,36]. It was assumed that the interfacial fracture energy remained stable with the efficient long exposure duration. To evaluate the effects of thermal cycles in water on the interfacial fracture energy, the following relation was proposed:

(9) $$\alpha_{C}=\frac{G_{f}^{\infty }}{G_{f}^{c}},$$

where $\alpha_{C}$ is the degradation coefficient of the interfacial fracture energy, and $G_{f}^{\infty }$ and $G_{f}^{c}$ are the interfacial fracture energy of aged specimens with efficient exposure duration and the control specimens, respectively. In the present case of all specimens exposed to thermal cycles in water, the lowest limit value was assumed to be 0.7. It is worth noting that the proposed degradation coefficient of the interfacial fracture energy is determined by the cases of the concrete compressive strength ranging from 25 MPa to 44 MPa.

## 4. Conclusions

This report investigated the influence of thermal cycles in air and in water on the bond behavior between a CFRP plate/sheet and concrete. Based on the experimental results, the following can be concluded:

1. Thermal cycles increase the interfacial fracture energy. The increase in the fracture energy of CFRP sheet–concrete is smaller than that of CFRP plate–concrete.
2. The failure modes of both CFRP sheet–concrete and CFRP plate–concrete are still concrete cohesive failure. The thermal cycles in water change the failure mode of both CFRP sheet–concrete and CFRP plate–concrete from concrete cohesive failure to interfacial debonding between the adhesive layer and concrete.
3. The degradation of the interfacial bond mainly results from water moisture with 270 thermal cycles in water. The internal stress induced by thermal cycles in air has insignificant effects on the interfacial fracture energy.
4. A reduction factor (0.7) for a CFRP–concrete structure for thermal cycles in water was proposed.

Based on the preceding conclusions, it is clear that the parameter of thermal cycles in water is one of the key environmental durability issues, which affects the bond between CFRP and concrete. Therefore, relevant consideration should be made during the design stage to ensure the safety and longevity of the structure. The proposed reduction factor only considered the specific range of compressive strength. In future research, the high concrete compressive strength should be taken into account in the reduction factor. Besides, a molecular dynamics model has been modeled to investigate the mechanism of the deterioration of the bond between SiO_2_ (concrete) and epoxy exposed to thermal cycles in water.

## Figures and Tables

**Table 1:** Constituent materials of carbon fiber reinforced polymer (CFRP)–concrete.

Materials	CFRP Plate–Concrete	CFRP Sheet–Concrete
Primer	Epoxy	Epoxy
Resin	-	Epoxy
Adhesive	Adhesive	-
Concrete	$f_{c}^{\prime }$ = 42.1 MPa	$f_{c}^{\prime }$ = 42.1 MPa

**Table 2:** Diffusivity properties of the epoxy and adhesive.

Parameter	Epoxy	Adhesive
Diffusivity coefficient (×10^−8^ mm^2^/s)	6.0	7.0
Equilibrium moisture (%)	2.40	3.53

**Table 3:** Diffusivity properties of CFRP.

Parameter	CFRP Sheet	CFRP Plate
Fiber volume fraction	0.3	0.67
Diffusivity coefficient (×10^−8^ mm^2^/s)	2.3	0.5
Equilibrium moisture (%)	1.68	0.8

**Table 4:** Elastic modulus of the constituent materials (GPa).

Materials	Control	Cov.	In Air	Cov.	In Water	Cov.
Epoxy	3.2	0.07	3.6	0.16	2.9	0.04
Adhesive	3.5	0.10	3.7	0.30	3.0	0.12
CFRP sheet	241.2	5.2	250.3	9.8	218.9	8.4
CFRP plate	176.5	3.5	178	8.3	173.0	5.1

**Table 5:** Details of specimens and pullout bond test results.

Specimens ^a^	No. Cycles	Conditioning	Failure Mode	*B* (mm^−1^)	*A* (με)	*P*_u_ (kN)	*G_f_* (N/mm)	*L_e_* (mm)
CSC-1	0	-	Concrete	27	3693	7.6	0.56	84
CSC-2	0	-	Concrete	32	3222	6.6	0.42	109
CSCA-1	270	Air	Concrete	40	3784	5.1	0.59	98
CSCA-2	270	Air	Concrete	46	5336	5.8	1.17	150
CSCW-1	270	Water	Interface	78	2476	7.7	0.25	87
CSCW-2	270	Water	Interface	39	2849	10.9	0.33	62
CPC-1	0	-	Concrete	22	2622	16.2	0.85	-
CPC-2	0	-	Concrete	22	2339	14.4	0.67	-
CPC-3	0	-	Concrete	15	3070	18.9	1.16	131
CPC-4	0	-	Concrete	42	2462	15.2	0.75	176
CPC-5	0	-	Concrete	33	2471	15.2	0.75	146
CPCA-1	270	Air	Concrete	18	2954	18.2	1.08	149
CPCA-2	270	Air	Concrete	26	2800	17.2	0.97	152
CPCW-1	270	Water	Interface	34	2415	14.9	0.72	146
CPCW-2	270	Water	Interface	36	2339	14.4	0.67	168

^a^ CSC–CFRP sheet–concrete. CPC–CFRP plate–concrete. A—270 thermal cycles in air. W—270 thermal cycles in water.

**Table 6:** Shear test data under the thermal cycles in air or water collected from the literature.

Source of Data	Specimen	FRP Type	No. Cycles	Exposure	$f_{c}^{\prime }$ (MPa)	*E*_f_*t*_f_ (GPa/mm)	*G*_f_ (N/mm)
Kabir et al. (2016) [10]	Control	Sheet	0	-	36.6	52.9	1.57
	CT2	Sheet	35	Air	36.6	52.9	1.99
	CT3	Sheet	90	Air	36.6	52.9	1.93
	CT4	Sheet	335	Air	36.6	52.9	1.65
Pierluigi Colombi et al. (2010) [18]	P18A	Plate	0	-	25.0	211.9	0.67
	P20A	Plate	0	-	25.0	211.9	0.50
	P2A	Plate	100	Water	24.9	211.9	0.55
	P4A	Plate	200	Water	24.7	211.9	0.64
	P6A	Plate	200	Water	24.7	211.9	0.78
Subramaniam et al. (2008) [34]	0 cycles	sheet	0	-	38.0	39.1	1
	100 cycles	sheet	100	Water	38.0	41.8	0.92
	200 cycles	sheet	200	Water	38.0	39.7	0.88
	300 cycles	sheet	300	Water	38.0	41.5	0.83
Xian et al. (2018) [5]	F-T/W-0-1	Plate	0	-	44.1	246.4	1.1
	F-T/W-0-2	Plate	0	-	44.1	246.4	0.78
	F-T/W-0-3	Plate	0	-	44.1	246.4	0.96
	F-T-M-30-1	Plate	30	90% RH	39.9	246.4	0.78
	F-T-M-30-2	Plate	30	90% RH	39.9	246.4	0.58
	F-T-M-60-1	Plate	60	90% RH	38.6	246.4	0.64
	F-T-M-60-2	Plate	60	90% RH	38.6	246.4	0.88
	F-T-M-90-1	Plate	90	90% RH	41.2	246.4	0.62
	F-T-M-90-2	Plate	90	90% RH	41.2	246.4	0.88
	F-T-M-90-3	Plate	90	90% RH	41.2	246.4	0.65
Gamage et al. (2015) [11]	A1-1	sheet	0	90% RH	30	40.5	0.78
	A1-2	sheet	0	90% RH	30	40.5	0.79
	A2-1	sheet	44	90% RH	30	40.5	0.62
	A2-2	sheet	44	90% RH	30	40.5	0.65
	A4-4	sheet	81	90% RH	30	40.5	0.59
	A4-5	sheet	81	90% RH	30	40.5	0.53
	A4-6	sheet	81	90% RH	30	40.5	0.62
	A4-7	sheet	81	90% RH	30	40.5	0.52
	A4-8	sheet	81	90% RH	30	40.5	0.54
	A9-9	sheet	312	90% RH	30	40.5	0.43
	A9-10	sheet	312	90% RH	30	40.5	0.48
	A11	sheet	600	90% RH	30	40.5	0.41
